# The 341C/T polymorphism in the GSTP1 gene is associated with increased risk of oesophageal cancer

**DOI:** 10.1186/1471-2156-11-47

**Published:** 2010-06-11

**Authors:** Dongping Li, Collet Dandara, M Iqbal Parker

**Affiliations:** 1 International Centre for Genetic Engineering and Biotechnology (ICGEB), UCT Campus , Anzio Road Observatory 7925, Cape Town, South Africa; 2 Division of Medical Biochemistry, Faculty of Health Science, University of Cape Town, Anzio Road, Observatory 7925, Cape Town, South Africa; 3 Division of Human Genetics, Faculty of Health Science, University of Cape Town, Anzio Road, Observatory 7925, South Africa

## Abstract

**Background:**

The Glutathione S-transferases (GSTs) comprise a group of enzymes that are critical in the detoxification of carcinogens. In this study the effects of polymorphisms in these genes on the risk of developing oesophageal squamous cell carcinoma (OSCC) were evaluated in a hospital-based case-control study in two South African population groups. Genetic polymorphisms in GSTs were investigated in 245 patients and 288 controls samples by PCR-RFLP analysis.

**Results:**

The *GSTP1 341T *variant was associated with significantly increased risk of developing OSCC as observed from the odds ratios for the *GSTP1 341C/T *and GSTP1 341T/T genotypes (OR = 4.98; 95%CI 3.05-8.11 and OR = 10.9; 95%CI 2.43-49.1, respectively) when compared to the homozygous GSTP1 341C/C genotype. The risk for OSCC in the combined GSTP1 341C/T and T/T genotypes was higher in tobacco smokers (OR = 7.51, 95% CI 3.82-14.7), alcohol consumers (OR = 15.3, 95% CI 1.81-12.9) and those using wood or charcoal for cooking and heating (OR = 12.1, 95% CI 3.26-49) when compared to those who did not smoke tobacco, or did not consume alcohol or user other forms of fuel for cooking and heating. Despite the close proximity of the two GSTP1 SNPs (313A>G and 341C>T), they were not in linkage disequilibrium in these two population groups (D':1.0, LOD: 0.52, r^2^: 0.225). The GSTP1 313A/G polymorphism on the other hand, did not display any association with OSSC. The homozygous GSTT1*0 genotype was associated with increased risk of OSCC (OR = 1.71, 95%CI 1.18-2.46) while the homozygous GSTM1*0 genotype was associated with significantly decreased risk of OSCC in the Mixed Ancestry subjects (OR= 0.39, 95%CI 0.25-0.62).

**Conclusions:**

This study shows that the risk of developing OSCC in the South African population can be partly explained by genetic polymorphisms in GST coding genes and their interaction with environmental factors such as tobacco smoke and alcohol consumption.

## Background

Oesophageal squamous cell carcinoma (OSCC) is the second most common cancer among African males in South Africa [1,2]. Although very little is known about the aetiology of OSCC in this population, several risk factors such as tobacco smoking, alcohol consumption and the prolonged use of wood or charcoal as sources of fuel for cooking and heating (resulting in excessive smoke inhalation), have generally been implicated [3,4]. Somatic mutations in the human pro-collagen genes [5], genetic polymorphisms in the androgen receptor gene [6], or genes coding for phase I and phase II detoxification enzymes [7-9], exposure to aflatoxin-, and fumonisin-contaminated maize, human papilloma virus (HPV) infection [10] and a habit of regular forced vomiting have all been proposed as major risk factors for OSCC among South Africans. Recent data imply that the environmental risk factors may be modified by polymorphisms in the carcinogen metabolizing genes i.e. gene-environment interactions [7].

The glutathione S-transferase (GST) family of enzymes play an important role in the detoxification of carcinogens by catalyzing the conjugation of glutathione (GSH) to electrophilic compounds [11-14]. Multiple tissue-specific GST isoforms accommodate a diverse range of substrates, thus conferring tissue specificity in the handling of certain carcinogens. Although there is evidence for the role of genetic polymorphisms in the alpha (A), mu (M), theta (T) and pi (P) GST gene families in a number of cancers [15-19], the current study investigated the role of the latter three in OSCC among South Africans because of their biological relevance in the metabolism of known carcinogens, allelic frequency and implications in previous epidemiological studies on cancer [15-19].

GSTM1 is principally expressed in the liver, with low levels in extra hepatic tissues. Genetic polymorphisms in the gene are due to either gene deletion (giving rise to GSTM1*0) or a single nucleotide change 534 C/G (causing the replacement of lysine 172 by aspartic acid) resulting in two alleles GSTM1*A and GSTM1*B, whose gene products do not show any differences in activity [13,14]. The GSTM1*0 occurs at different frequencies in different populations: 19%-33% in Africans [15-17], 30%-52% among Caucasians [18,19] and 55% among Asians [20].

GSTT1 on the other hand, is expressed at high levels in extra hepatic tissues, including the kidney, liver and the gastrointestinal tract, suggesting an important role in the protection against carcinogens and other xenobiotics in these tissues [13,21,22]. Two GSTT1 variants have been identified, one is an entire gene deletion (referred to as GSTT1*0) [23] and the second is a single base change, 310 A/T (referred to as GSTT1*B) which is associated with abolished GSTT1 activity [24]. There is a clear ethnic variation in the distribution of the homozygous GSTT1 null genotype occurring in 10%-26% Africans and Caucasians [15-18] and 55%-75% in Koreans, Japanese and Chinese [20,25,26].

GSTP1 is the major GST expressed in extra hepatic tissues such as the lungs and the oesophagus with very little expression in the liver [13,21,22] and has been shown to be over expressed in several malignant tissues compared to their matched normal tissues [27]. Two single nucleotide polymorphisms (SNPs) in GSTP1 resulting in amino acid substitutions that affect enzyme activity function are rs1695 (formerly rs947894 which is due to an A313G substitution resulting in an Ile105Val amino acid change) and rs1138272 (formerly rs1799811 which is due to a C341T substitution resulting in an Ala114Val amino acid change) [28-30]. The *GSTP1 313G *variant has been widely studied and occurs at frequencies of 14%-20% among Black Africans [15,16], 28%-32% among Caucasians [31] and 14-18% among Asians [29,32]. Very few studies have investigated the role of the *GSTP1 341T *variant in the development of OSCC. We investigated the role of the GSTM1, T1 and P1 polymorphisms in OSCC because of the conflicting reports on their role in gastric, breast and lung cancers [33-36]. Our data suggest that the *GSTP1 341T *variant significantly predisposes individuals to OSCC.

## Results

### Distribution of GSTP1 alleles and genotypes

The distribution of the *GSTP1 313 A/G *polymorphism was not significantly different between patients and controls among either the Black or the Mixed Ancestry groups. The GSTP1 313G variant occurred with a frequency of 39% in patients vs. 37% in controls in the Black subjects and 38% in patients vs. 41% in controls in the Mixed Ancestry subjects. In contrast, the distribution of the *GSTP1 341C/T *polymorphism differed between patients and controls with the *GSTP1 341T *variant occurring at frequencies of 22% in patients vs. 7% in controls in the Black population and 19% in patients vs. 3% in controls in those of Mixed Ancestry (Table 1). In both the Black and the Mixed Ancestry subjects, the *GSTP1 341T *variant was associated with significantly increased risk of OSCC as observed from the heterozygous *GSTP1 341C/T *and homozygous *GSTP1 341T/T *genotypes which were associated with increased risk for developing OSCC among both the Black African and Mixed Ancestry subjects (OR = 4.98; 95%CI 3.05-8.11 and OR = 10.9; 95%CI 2.43-49.1, respectively, for each genotype). Odds ratios for each racial group separately are provided in additional file 1.

**Table 1:** The distribution of the GST genotypes in Black and Mixed Ancestry in South Africans

Genotypes	Black African subjects		Mixed Ancestry subjects		Analysis of subjects combined	
	Patients N (%)	Controls N (%)	Patients N (%)	Controls N (%)	**OR**^**1 **^**(95%CI) *p*-*value***	**OR**^2 ^**(95%CI) *p-value***
GSTT1^a^						
*1	57 (40)	109 (59)	68 (68)	69 (73)	1.00	1.00
*0 (rs71748309)	84 (60)	77 (41)	29 (29)	25 (27)	1.55 (1.09-2.21) 0.014	1.71 (1.18-2.46) 0.004
GSTP1 313 A/G (Ile105Val) (rs1695)						
*313A/A*	56 (40)	76 (41)	34 (34)	30 (32)	1.00	
*313A/G*	59 (42)	83 (45)	52 (52)	51 (54)	0.96 (0.66-1.40) 0.846	1.01 (0.68-1.48) 0.973
*313G/G*	26 (18)	27 (14)	11 (11)	13 (14)	1.08 (0.64-1.82) 0.786	1.21 (0.71-2.07) 0.490
*Test for deviation from Hardy-Weinberg Equilibrium*						
*χ*^2^	*2.139*	*0.360*	*1.874*	*1.407*		
*p*	*0.144*	*0.549*	*0.177*	*0.236*		
*313A freq*	*0.61+0.03*	*0.63+0.02*	*0.62+0.03*	*0.59+0.03*		
*313G freq*	*0.39+0.03*	*0.37+0.02*	*0.38+0.03*	*0.41+0.03*		
GSTP1 341 C/T (Ala114Val) (rs1138272)						
*341C/C*	85 (60)	163 (88)	65 (65)	89 (95)	1.00	1.00
*341C/T*	49 (35)	21 (11)	27 (27)	5 (5)	4.98 (3.05-8.11) 0.001	5.05 (3.06-8.32) 0.001
*341T/T*	7 (5)	2 (1)	5 (5)	0 (0)	10.9 (2.43-49.1) 0.002	9.58 (2.08-44.0) 0.004
Test for deviation from Hardy-Weinberg Equilibrium						
*χ^2^*	*0*	*1.397*	*0.876*	*0.137*		
*p*	*0.986*	*0.237*	*0.349*	*0.712*		
*341C freq*	*0.78+0.02*	*0.93+0.01*	*0.81+0.03*	*0.97+0.01*		
*341T freq*	*0.22+0.02*	*0.07+0.01*	*0.19+0.03*	*0.03+0.01*		

OR^1^, odds ratio not adjusted for confounders; OR^2^, odds ratio adjusted for the following confounding variables, age, sex, race, tobacco smoking, alcohol consumption and use of wood or charcoal; . ^a ^*1 signifies carrier of gene (either homozygous or heterozygous carrier), *0 signifies homozygous gene deletion

Of the 116 subjects who were either homo- or heterozygous for the *GSTP1 341C/T *polymorphism 76% (n = 88) were patients while only 24% (n = 28) were controls. It is important to note that this report is one of very few studies on the *GSTP1 341C/T *polymorphism in OSCC since most published studies focus on the *GSTP1 313A/G *polymorphism. Using linkage format in HaploView to calculate linkage between GSTP1 313A/G and GSTP1 341C/T SNPs the following parameters were observed, D':1.0, LOD: 0.52 and r^2^: 0.225. The observed haplotypes were AT (0.555), CG (0.202), GT (0.159) and AC (0.084). Analyses of the stratification between GSTP1 and tobacco smoking habits, alcohol consumption and combustion of charcoal or wood as fuel, the *GSTP1 341C/T+341T/T *combined genotypes were associated with a higher risk for OSCC among tobacco smokers (OR = 7.51, 95%CI 3.82-14.7), alcohol consumers (OR = 15.3, 95%CI 1.81-12.9) and among users of charcoal or wood for fuel (OR = 12.1, 95% CI 3.26-49) when compared to non-smokers (OR = 5.31, 95% CI 2.58-27.6), non-alcohol consumers (OR = 10.9, 95% CI 4.09-28.9) and users of other forms of fuel for cooking and heating (OR = 4.93, 95% CI 2.47-9.84) (Table 2). When measures of biological interaction were calculated the *GSTP1 341C/T+341T/T *combined genotype was associated with high relative risk for OSCC only among users of wood/charcoal (RR = 29.9) and tobacco smokers (RR = 4.28) while GSTM1*0/*0 genotype interacting with smoking was surprisingly associated with reduced relative risk (RR = 0.364) (Table 3 and Additional file 2). Additional file 2 shows the relative risk with contributions from *GSTP1 341C/T+341T/T *combined genotype and exposure to either use of wood/charcoal as fuel for heating and cooking and GSTM1*0/*0 interacting with tobacco smoking, respectively In all the above statistical analyses on the genotype distribution and interaction variables the parameters were adjusted for each other (tobacco smoking, alcohol consumption, race, age, gender and use of wood or charcoal). Interestingly, all wood or charcoal users with the GSTP1 341T/T genotype (results not shown) were patients and of the 15 subjects who had the *GSTP1 341T/T *genotype, 14 (93%) were smokers of whom, 13 were patients.

**Table 2:** Interactions between GST genotypes, alcohol consumption, tobacco smoking and fuel source among oesophageal cancer patients and controls

Genotypes	Patients N(%)	Controls N(%)	OR (95%CI) p-value	Patients N(%)	Controls N (%)	OR (95%CI) p-value
	***Tobacco smokers***^a^			***Non smokers***^a^		
GSTT1*1	100 (56)	112 (61)	1.00	27 (43)	66 (69)	1.00
GSTT1*0	77 (44)	73 (39)	1.01 (0.62-1.66) 0.951	36 (57)	29 (31)	2.92(1.04-8.16) 0.042
GSTM1*1	151 (85)	125 (68)	1.00	55 (87)	75 (79	1.00
GSTM1*0	125 (15)	60 (32)	0.30 (0.16-0.56) 0.001	8 (13)	20 (21)	0.46 (0.12-1.81) 0.269
GSTP1 313A/A	71 (40)	77 (42)	1.00	21 (33)	30 (32)	1.00
GSTP1 313 A/G	78 (44)	86 (46)	0.96 (0.58-1.61) 0.898	33 (52)	48 (50)	0.53 (0.18-1.51) 0.233
GSTP1 313G/G	28 (16)	22 (12)	1.54 (0.74-3.21) 0.250	9 (14)	17 (18)	0.44 (0.10-1.93) 0.278
GSTP1 341 C/C	106 (60)	166 (90)	1.00	44 (70)	86 (91)	1.00
GSTP1-341C/T + GSTP1-341T/T	71 (40)	19 (10)	7.51 (3.82-14.7) 0.001	19 (30)	9 (9)	5.31 (2.58-27.6) 0.001
	**Alcohol consumers**^b^			**Non consumers**^b^		
GSTT1*1	89 (54)	101 (60)	1.00	38 (51)	77 (68)	1.00
GSTT1*0	76 (46)	66 (40)	1.03 (0.60-1.77) 0.911	37 (49)	36 (32)	1.66 (0.77-3.59) 0.197
GSTM1*1	143 (87)	123 (74)	1.00	63 (84)	77 (68)	1.00
GSTM1*0	22 (13)	44 (26)	0.33 (0.16-0.66) 0.002	12 (16)	36 (32)	0.27 (0.10-0.74) 0.011
GSTP1 313A/A	63 (38)	74 (44)	1.00	29 (39)	33 (29)	1.00
GSTP1 313 A/G	80 (48)	75 (45)	1.22 (0.70-2.13) 0.474	31 (41)	58 (52)	0.41 (0.17-0.96) 0.039
GSTP1 313G/G	22 (13)	18 (11)	1.69 (0.72-3.95) 0.226	15 (20)	22 (19)	0.64 (0.24-1.76) 0.394
GSTP1 341 C/C	105 (64)	148 (89)	1.00	45 (60)	104 (92)	1.00
GSTP1-341C/T + GSTP1-341T/T	60 (36)	19 (11)	15.3 (1.81-12.9) 0.012	30 (40)	9 (8)	10.9 (4.09-28.9) 0.001
	**Wood or charcoal users**^c^			**Non users**^c^		
GSTT1*1	54 (61)	25(58)	1.00	52 (60)	153 (65)	1.00
GSTT1*0	34 (39)	18 (42)	0.77 (0.35-1.68) 0.510	35 (40)	84 (35)	1.67 (0.94-2.93) 0.080
GSTM1*1	72 (82)	27 (63)	1.00	77 (89)	173 (73)	1.00
GSTM1*0	16 (18)	16 (37)	0.41 (0.17-0.96) 0.041	10 (11)	64 (27)	0.26 (0.12-0.56) 0.001
GSTP1 313A/A	38 (43)	17 (40)	1.00	34 (39)	90 (38)	1.00
GSTP1 313 A/G	34 (39)	23 (53)	0.71 (0.30-1.69) 0.441	41 (47)	110 (46)	0.97 (0.55-1.73) 0.922
GSTP1 313G/G	16 (18)	3 (7)	3.28 (0.79-13.7) 0.103	12 (14)	37 (16)	1.11 (0.48-2.53) 0.809
GSTP1 341 C/C	42 (48)	40 (93)	1.00	57 (66)	212 (89)	1.00
GSTP1-341C/T + GSTP1-341T/T	46 (52)	3 (7)	12.1 (3.26-49.0) 0.001	30 (34)	25 (11)	4.93 (2.47-9.84) 0.001

*1 signifies carrier of gene, *0 signifies homozygous gene deletion. Odds ratios adjusted for ^a^alcohol consumption, race, sex, age, wood or charcoal use; ^b^tobacco smoking , race, sex, age, wood or charcoal use; ^c^alcohol consumption, race, sex, age, tobacco smoking

**Table 3:** Calculating measures of biological interaction between genotypes and environmental exposures

Dichotomous risk factors		RR	95% CI		RERI	AP	S
**Risk 1**^**a**^**(Genotypes)**	**Risk 2**		**lower**	**upper**			
GSTT1*0/*0	alcohol	2.158	1.175	3.965	-0.322	-0.149	0.783
GSTT1*0/*0	smoking	1.771	0.958	3.274	-1.559	-0.880	0.331
GSTT1*0/*0	wood/charcoal	2.738	1.443	5.198	0.213	0.078	1.140
GSTM1*0/*0	alcohol	0.399	0.165	0.760	0.058	0.145	0.912
GSTM1*0/*0	wood/charcoal	1.075	0.508	2.276	-1.641	-1.527	0.044
GSTM1*0/*0	smoking	0.364	0.190	0.699	-0.148	-0.407	1.304
*GSTP1 313 A/G +G/G*	alcohol	0.922	0.488	1.743	0.597	0.647	0.115
*GSTP1 313A/G +G/G*	smoking	0.779	0.390	1.553	0.113	0.145	0.663
*GSTP1 313A/G +G/G*	wood/charcoal	1.738	0.978	3.087	0.185	0.107	1.335
*GSTP1 341C/T+T/T*	alcohol	6.321	3.054	13.09	-0.514	-0.081	0.912
*GSTP1 341C/T+T/T*	smoking	4.284	2.158	8.507	1.032	0.241	1.458
*GSTP1 341C/T+T/T*	wood/charcoal	29.87	8.968	99.49	27.38	0.917	19.40

^a^The references for the interaction exposures were; GSTM1*1 + non-exposure, GSTT1*1 + non-exposure, *GSTP1 313 A/A *+ non-exposure, *GSTP1 341 C/C *+ non-exposure. Where non exposure refers to any of the following; non-smoker, non-alcohol consumer, and not exposed to use of wood/charcoal. The interaction model used could only factor in two dichotomous risk factors. Thus the following genotype combinations were used; GSTM1 (GSTM1*1/*1 vs. GSTM1*0/*0); GSTT1 (GSTT1*1/*1 vs. GSTT1*0/*0); *GSTP105 (A/A *vs. *A/G+GG)*; *GSTTP114 (C/C *vs. *C/T+T/T)*. For each interaction, four disjoint categories were created. For example, in the case of smoking and GSTP114 (non-smoker and C/C, non-smoker and C/T+TT, smoker and C/C, smoker and *C/T+T/T*). RR= relative risk; RERI = the relative excess risk due to interaction; AP = the attributable risk due to interaction; S = the synergy index.

### GSTM1 and T1 frequency distribution

Using logistic regression analysis, the effects of homozygous GSTM1 deletion (rs4025935) and GSTT1 gene deletion (rs71748309) were calculated after adjusting for sex (our previous paper had shown a 2-fold risk among men), race (due to the observed differences in the allele frequencies in the Black and Mixed Ancestry subjects), tobacco smoking, alcohol consumption and age. The homozygous GSTT1*0 genotype was associated with significantly increased risk of OSCC (OR = 1.71, 95% CI 1.18-2.46) while the homozygous GSTM1*0 genotype was associated with significantly decreased risk of OSCC (OR = 0.39, 95% CI 0.25-0.62). These results support our earlier observations with the CYP3A5 and SULT1A1 gene polymorphisms [7,8].

### Combinations of GSTM1, T1 and P1 genotypes

The expression of human GSTs shows unique tissue specificity; thus, tissues that express a variety of GSTs are probably more effective in detoxifying a wide range of carcinogens. We evaluated the effects of having certain GST genotype combinations on the risk for OSCC and used a logistic model to take into account the contribution of other confounders (Table 4). All the genotype combinations were analysed after adjusting for the covariates which had shown an effect on oesophageal cancer when we applied the maximum likelihood probit estimation (Probit estimates: number of observations = 535, LR Chi^2^(10) = 257.64, P > Chi^2^= 0.0001). Having the *GSTP1 341C/T+341 T/T *genotype in combination with the homozygous GSTT1*0 genotype was associated with a higher risk of OSCC (OR = 14.9, 95% CI 5.64-39.4) when compared to the GSTP1 341C/T+341T/T in combination with the GSTT1*1 genotype (OR = 3.76, 95% CI 1.82-7.77) (Table 4). Furthermore, all the subjects with a combination of the homozygous *GSTP1 341T/T *genotype and the GSTT1*0 genotype (n = 3) were patients (data not shown). This combination represents severely reduced detoxification capacity due to deletion of GSTT1 and the selective activity of the *GSTP1 341T *variant protein, thus confirming the important role of these GSTs in detoxification. Interpretation of significance was corrected using the Bonferroni correction such that significance in the interaction models in Table 4 was only noted when the P value was < 0.025.

**Table 4:** Comparison of the risk of SCC of the oesophagus associated with different GST genotypes

Genotypes	Patients N(%)	Controls N(%)	OR (95%CI) *p-value *	Patients N(%)	Controls N(%)	OR (95%CI) *p-value *
	GSTM1*0			GSTM1*1		
GSTM1*1	98 (87)	65 (64)	1.00	108 (85)	135 (76)	1.00
GSTM1*0	15 (13)	37 (36)	0.27 (0.12-0.62) 0.002	19 (15)	43 (24)	0.36 (0.17-0.76) 0.008
GSTP1 313A/A	49 (43)	38 (37)	1.00	43 (34)	69 (39)	1.00
GSTP1 313 A/G	49 (44)	50 (49)	0.51 (0.25-1.05) 0.069	62 (49)	84 (47)	1.28 (0.69-3.37) 0.434
GSTP1 313G/G	15 (13)	14 (14)	0.42 (0.13-1.33) 0.139	22 (17)	25 (14)	2.36 (1.04-5.37) 0.041
GSTP1 341 C/C	70 (62)	94 (92)	1.00	80 (63)	158 (89)	1.00
GSTP1-341C/T + GSTP1-341T/T	43 (38)	8 (8)	14.9 (5.64-39.4) 0.001	47 (37)	20 (11)	3.76 (1.82-7.77) 0.001
	GSTM1*0			GSTM1*1		
GSTT1*1	19 (56)	43 (54)	1.00	108 (52)	135 (68)	1.00
GSTT1*0	15 (44)	37 (46)	0.73 (0.25-2.10) 0.559	98 (48)	65 (32)	1.55 (093-2.58) 0.093
GSTP1 313A/A	13 (38)	32 (40)	1.00	79 (38)	75 (37)	1.00
GSTP1 313 A/G	15 (44)	39 (49)	1.63 (0.52-5.12) 0.40	96 (47)	95 (48)	0.80 (0.47-1.36) 0.417
GSTP1 313G/G	6 (18)	9 (11)	2.78 (0.59-13.0) 0.193	31 (15)	30 (15)	1.04 (0.50-2.19) 0.914
GSTP1 341 C/C	22 (65)	73 (91)	1.00	128 (62)	179 (90)	1.00
GSTP1-341C/T + GSTP1-341T/T	12 (35)	7 (9)	6.06 (1.79-20.5) 0.004	78 (38)	21 (10)	6.31 (3.26-12.2) 0.001

OR, odds ratios adjusted for age, sex, race, tobacco smoking, alcohol consumption and use of charcoal or wood for cooking. *1 signifies carrier of gene, *0 signifies homozygous gene deletion.

Interestingly, of the 15 subjects homozygous for the *GSTP1 341T/T *genotype, 80% (n = 12) had the GSTT1 wild type genotype (data not shown). Furthermore, none of the subjects had a combination of the GSTM1*0 genotype + homozygous *GSTP1 341T/T *genotype; Thus, all the subjects (n = 15) with the homozygous *GSTP1 341T/T *genotype were GSTM1 positive and 87% of them (n = 13) were OSCC patients, possibly pointing to compensation between genes such that, in the absence of GSTM1 (the case of deletion) there is most likely to be another normally functioning GST gene whose product participates in the same metabolic pathway (in this case GSTP1 341C/C).

## Discussion

The disproportionate geographical distribution of OSCC can be attributed to differences in environmental exposures while the variability observed amongst residents of the same locality can be attributed mainly to differences in genetic predisposition and to host defence mechanisms. Most environmental carcinogens require prior metabolic activation in order to elicit their effects but many of the enzymes involved in carcinogen metabolism exhibit genetic polymorphisms resulting in variability in both their level of expression [37] and activity [38,39]. This study investigated the role of such polymorphic variants in the glutathione S-transferase genes because of their involvement in the detoxification of many carcinogens as one of the major phase II enzymes. Although multiple forms of GSTs can accommodate diverse substrates, the distribution of the GST isoforms varies between different tissues, suggesting potential differences in the manner in which these tissues are detoxified [13,21].

The observation that tobacco smokers, alcohol consumers or those using wood or charcoal for cooking and heating carrying the GSTP1 341T variant in either the heterozygous or homozygous form had a higher risk of developing OSCC might be due to decreased detoxification of carcinogens as a result of decreased activity of the GSTP1 variant or changed substrate specificity [7,28,30,31]. The higher incidence of OSCC among tobacco smokers who either had the heterozygous *GSTP1 341C/T *genotype or homozygous *GSTP1 341T/T *genotype clearly indicates a gene-environment interaction. This is the first study showing an association between genetic polymorphism in GSTP1 and OSCC in the South African population.

Of all the GST enzymes, GSTP1 has the highest specific activity towards benzo (α) pyrene diol epoxide and benzo (α) pyrene-7ß, α hydrodiol-9 α,10 α-epoxide (BPDE), the major metabolites of benzo (α) pyrene [30]. Benzo (α) pyrene and its metabolites are some of the major components of cigarette smoke [40] and are also detected in wood or charcoal combustion under conditions of limited oxygen supply. GSTP1 is predominantly expressed in extra hepatic tissues including the oesophagus and is therefore likely to play a major role in the detoxification of carcinogens that are activated within these tissues [13,21,22].

The GSTM1*0 genotype on the other hand, was generally associated with decreased risk of OSCC. Our observation for GSTT1*0 and GSTM1*0 is in agreement with other findings in the Caucasian populations [41] and contrary to the report by Anantharaman *et al*. [42] who observed an inverse correlation, i.e. reduced risk of OSCC among Indian carriers of the GSTT1*0 genotype and increased risk among GSTM1*0 carriers. The above differences could be due to the type of environmental exposures, quantity of exposure, method of exposure and the tissue distribution of the GST enzymes [13,22]. The widely studied GSTP1 polymorphism, *GSTP1 313A/G*, was not associated with any risk for OSCC in any of the population groups in our study. The observed risks associated with the *GSTP1 313A/G *in other studies could be due to linkage disequilibrium between this polymorphism and the *GSTP1 341C/T *polymorphism, a phenomenon which was not manifested in the South African population when one considers the linkage parameters calculated using HaploView (D': 1.0, LOD:0.52, r2: 0.225).

The variation in the impact of GSTM1, GSTT1 and GSTP1 in oesophageal cancer susceptibility could be due to their differences in organ localization and metabolic functions [11,13,21,22]. The differences observed across different ethnic groups in different geographical areas could be due to differences in major exposure variables and environmental interactions. The above is even more plausible in the case of GSTP1 in which the allelic variants have been shown to differentially and preferentially metabolize bulky substrates compared to the wild type allele [30,31]. It should be noted that our results may not be able to satisfy the power requirements for gene frequencies that are less than 15%.

## Conclusions

We have shown that the risk of developing OSCC in the South Africa population can be partly explained by interactions between genetic polymorphisms in the GST genes and environmental factors such as tobacco smoking and alcohol consumption. The GSTP1 341 C/T polymorphism was associated with the higher risks for OSCC among subjects exposed to potential sources of carcinogens.

## Methods

### Study subjects

The general design of the study has been as previously described [7-9]. Briefly, all the patients (n = 245) were diagnosed with histologically confirmed OSCC at Groote Schuur Hospital, Cape Town, South Africa. Controls (n = 288) were age, sex, and geographically-matched individuals to the patients but with no obvious sign of disease. Controls with prior history of cancer were excluded. The patients and controls were recruited between 1997 and 2003 and were the same as those reported in earlier studies [7-9]. The Black subjects were Xhosa-speaking South Africans who originally came from either the Eastern Cape or the Western Cape. The Mixed Ancestry (commonly referred to as “coloured”) subjects are a result of intermarriages between races including Black Africans, Western Europeans, the Khoisan, Indonesians, and Malaysians who settled in the Cape from the middle of the 17^th ^century. Written or informed consent was obtained before subjects were enrolled into the study. A questionnaire gathering details on demographics (age, sex, and race), smoking habits, alcohol consumption and family history of cancer was completed on all subjects by a trained interviewer. The classification of tobacco smokers and alcohol consumers was according to Dandara et al. [7]. Blood was collected from all participating subjects by a trained phlebotomist and processed for DNA isolation. This study was approved by the University of Cape Town, Human Ethics Research Committee.

Sample size was calculated using an online software by Daniel Soper http://www.danielsoper.com/statcalc/calc01.aspx, using alpha 0.05, six possible predictors of oesophageal cancer (race, tobacco smoking, alcohol consumption, race/ethnicity, age and use of charcoal or wood as fuel for cooking and heating), an anticipated minimum allele frequency of 0.15 for each of the gene variants or polymorphisms and desired 90% power. This meant that we required a minimum of 123 samples.

### GSTM1 and GSTT1 genotyping

Genotyping of the GSTT1 and GSTM1 alleles was performed by multiplex PCR using minor modifications of the method of Arand *et al*. [43] using human serum albumin (HSA) as an internal control. The primer pairs used in the amplification of GSTT1, GSTM1 and HSA were, 5'-TTC CTT ACT GGT CCT CAC ATC TC-3'/5'-TCA CCG GAT CAT GGC CAG CA-3', 5'-GAA CTC CCT GAA AAG CTA AAG C-3'/5'-GTT GGG CTC AAA TAT ACG GTG G-3'and 5'-GCC CTC TGC TAA CAA GTC CTA C -3'/5'-GCC CTA AAA AGA AAA TCC CCA ATC-3', respectively. PCR amplification generated 480 bp, 219 bp, and 350 bp fragments for GSTT1, GSTM1 and HSA, respectively. Each multiplex PCR reaction consisted of 100 ng genomic DNA, 1×PCR buffer containing 1.5 mM MgCl_2_, 0.2 mM dNTP mixture, 0.2 μM of GSTM1 primers, 0.3 μM of GSTT1 and HSA primers and 1 U *Taq *DNA polymerase (Biotaq™) in a final volume of 50 μl. The PCR consisted of an initial denaturation step at 94°C for 5 minutes, followed by 35 cycles of denaturation at 94°C for 1 minute, annealing at 62°C for 1 minute, elongation at 72°C for 1 minute and a final extension step at 72°C for 5 minutes. The amplified products were visualised by electrophoresis in ethidium-bromide-stained 2% agarose gel. Gene deletion was assumed as the absence of either or both of the GSTM1 or GSTT1 fragments in the presence of the HSA fragment.

### Detection of the GSTP1 single nucleotide polymorphisms, 313 A/G and 341C/T

The genotyping of the GSTP1 gene for the detection of the single nucleotide changes, 313A/G (rs1695, GSTP1 Ile105Val) and 341C/T (rs1138272, Ala114Val) was done according to the method of Tan *et al*. [44]. The primer pairs used in the PCR amplification were, 5'-ACG CAC ATC CTC TTC CCC TC-3'/5'-TAC TTG GCT GGT TGA TGT CC-3'and 5'-CAA GGA TGG ACA GGC AGA ATG G -3'/5'-ATG GCT CAC ACC TGT GTC CAT C-3', respectively. Each PCR reaction contained 100 ng genomic DNA, 1×PCR buffer containing 1.5 mM MgCl_2_, 0.2 mM dNTP mixture, 0.2 μM of both primer set and 1 U *Taq *DNA polymerase (Takara or Invitrogen) in final volume of 50 μl. The PCR conditions for both reactions were as follows, denaturation step at 95°C for 3 minutes, followed by 30 cycles of denaturation at 95°C for 30 seconds, annealing at 57°C/63°C for 30 seconds, elongation at 72°C for 30 seconds and a final extension at 72°C for 7 minutes. The PCR products were digested using *Bsm *AI (wildtype = 440 bp, mutant = 212 bp + 228 bp) or *Aci *I (wild type = 172 bp + 195 bp, mutant = 367 bp) for the detection of the 313A/G or 341C/T base changes, respectively.

### Statistical analysis

The patient and control DNA samples were genotyped randomly without the researcher knowing whether they were working on patient or control DNA. STATA was used for the multivariate analysis and logistic regression in order to compare the distribution of the different variables between patients and controls, using odds ratios (OR) and the 95% confidence intervals (95%CI). Interactions between tobacco smoking, alcohol consumption, use of wood and GST genotypes were analyzed. The odds ratios were adjusted for sex and age. A p-value of <0.05 was considered statistically significant. There was no difference in statistical significance after Bonferroni Correction for multiple comparisons (i.e. reducing significance level to *p*= 0.017). Tests for deviation from Hardy-Weinberg Equilibrium were calculated using online program software on: http://ihg.gsf.de (under the link; Genotyping). This software is provided by the Institute of Human Genetics (Technical University Munich +Helmholtz Center Munich) German Research Center for Environmental Health. There was no deviation from Hardy- Weinberg among controls in the genotyping for the two GSTP1 single nucleotide polymorphisms.

### Calculation of measures of biological interaction

Measures of biological interaction were calculated from values obtained from logistic regression and covariance analysis using STATA according to Andersson et al. [45] to produce the output necessary for assessment of biological interaction using a model found on: http://www.epinet.se. The model calculates the following measures of biological interaction; RR, relative risk; RERI, the relative excess risk due to interaction; AP, the attributable proportion due to interaction; S, the synegy index (see Table 3).

## Authors' contributions

DL carried out most of the experiments as part of her PhD work; CD was co-supervisor of the PhD thesis and assisted with some of the experimental work and design of the project, MIP was the PhD supervisor, conceived and supervised the study. All authors read and approved the final manuscript.

## Supplementary Material

Additional file 1 **Table showing the distribution of the GST genotypes in Black and Mixed Ancestry in South Africans**. This Table shows the analysis of the GST genotype distributions in the two populations analysed separately, forming the basis of combined analysis. Click here for file

Additional file 2 **Graphs showing an example of relative risk with contributions from different exposure categories (gene-environment interaction)**. Representative graphs illustrating the interaction between GSTP1 341C/C+T/T genotype with wood/charcoal (graph A) and the interaction between GSTM1*0/*0 genotype with smoking (graph B). In both graphs, U represents is the reference baseline exposure which is GSTP1 341C/C genotype + none exposure to wood/charcoal in graph **A; **and GSTM1*1/*1 + being non-smoker in graph B. Calculations done according to according to Andersson et al.[39]. Click here for file
