# Supplementary material for: The 341C/T polymorphism in the GSTP1 gene is associated with increased risk of oesophageal cancer
Source: BMC Genet. 2010 Jun 11;11:47. doi: 10.1186/1471-2156-11-47 (PMC2891604; doi:10.1186/1471-2156-11-47)
Supplement: Additional file 1 — Table showing the distribution of the GST genotypes in Black and Mixed Ancestry in South Africans. This Table shows the analysis of the GST genotype distributions in the two populations analysed separately, forming the basis of combined analysis. [file 1471-2156-11-47-S1.DOCX]

**Additional file 1**: The distribution of the GST genotypes in Black and Mixed Ancestry in South Africans

| **Genotypes** | **Black subjects** | | **OR^#^ (95%CI) *p*-*value*** | **Mixed Ancestry subjects** | | **OR^#^ (95%CI) *p-value*** |
| --- | --- | --- | --- | --- | --- | --- |
|  | **Patients** | **Controls** |  | **Patients** | **Controls** |  |
|  | N (%) | N (%) |  | N (%) | N (%) |  |
| GSTT1^a^ | | | | | | |
| **1* | 57 (40) | 109 (59) | 1.00 | 68 (68) | 69 (73) | 1.00 |
| **0* | 84 (60) | 77 (41) | 2.26 (1.42-3.60) 0.001 | 29 (29) | 25 (27) | 1.29 (0.63-2.65) 0.488 |
| GSTM1^a^ | | | | | | |
| **1* | 117 (83) | 145 (78) | 1.00 | 87 (87) | 55 (59) | 1.00 |
| **0* | 24 (17) | 41 (22) | 0.69 (0.39-1.23) 0.208 | 10 (10) | 39 (41) | 0.10 (0.04-0.27) 0.001 |
| GSTP1 313 A/G (Ile105Val) | | | | | | |
| *313A/A* | 56 (40) | 76 (41) | 1.00 | 34 (34) | 30 (32) | 1.00 |
| *313A/G* | 59 (42) | 83 (45) | 0.96 (0.58-1.58) 0.874 | 52 (52) | 51 (54) | 1.17 (0.57-2.40) 0.670 |
| *313G/G* | 26 (18) | 27 (14) | 1.29 (0.66-2.49) 0.454 | 11 (11) | 13 (14) | 0.61 (0.20-1.88) 0.398 |
| *Test for deviation from Hardy-Weinberg Equilibrium* | | | | | | |
| *χ^2^* | *2.139* | *0.360* |  | *1.874* | *1.407* |  |
| *p* | *0.144* | *0.549* |  | *0.177* | *0.236* |  |
| *313A freq* | *0.61+0.03* | *0.63+0.02* |  | *0.62+0.03* | *0.59+0.03* |  |
| *313G freq* | *0.39+0.03* | *0.37+0.02* |  | 0.38+0.03 | 0.41+0.03 |  |
| GSTP1 341 C/T (Ala114Val) | | | | | | |
| *341C/C* | 85 (60) | 163 (88) | 1.00 | 65 (65) | 89 (95) | 1.00 |
| *341C/T* | 49 (35) | 21 (11) | 6.02 (3.18-11.4) 0.001 | 27 (27) | 5 (5) | 11.9 (3.78-37.6) 0.001 |
| *341T/T* | 7 (5) | 2 (1) | 9.60 (1.87-49.4) 0.007 | 5 (5) | 0 (0) | - |
| *Test for deviation from Hardy-Weinberg Equilibrium* | | | | | | |
| *χ^2^* | *0* | *1.397* |  | *0.876* | *0.137* |  |
| *p* | *0.986* | *0.237* |  | *0.349* | *0.712* |  |
| *341C freq* | *0.78+0.02* | *0.93+0.01* |  | *0.81+0.03* | *0.97+0.01* |  |
| *341T freq* | *0.22+0.02* | *0.07+0.01* |  | *0.19+0.03* | *0.03+0.01* |  |

#Odds ratios adjusted for age and sex. ^a^ *1 signifies carrier of gene, *0 signifies homozygous gene deletion.
