# Supplementary material for: The 341C/T polymorphism in the GSTP1 gene is associated with increased risk of oesophageal cancer
Source: BMC Genet. 2010 Jun 11;11:47. doi: 10.1186/1471-2156-11-47 (PMC2891604; doi:10.1186/1471-2156-11-47)
Supplement: Additional file 2 — Graphs showing an example of relative risk with contributions from different exposure categories (gene-environment interaction). Representative graphs illustrating the interaction between GSTP1 341C/C+T/T genotype with wood/charcoal (graph A) and the interaction between GSTM1*0/*0 genotype with smoking (graph B). In both graphs, U represents is the reference baseline exposure which is GSTP1 341C/C genotype + none exposure to wood/charcoal in graph A; and GSTM1*1/*1 + being non-smoker in graph B. Calculations done according to according to Andersson et al.[39]. [file 1471-2156-11-47-S2.DOC]

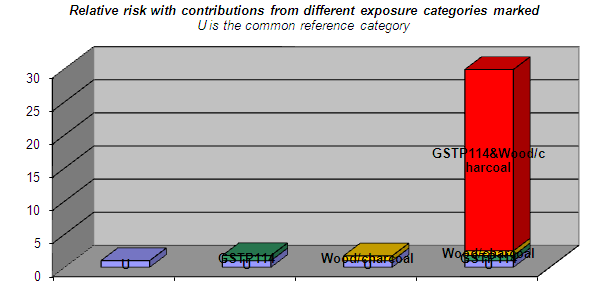


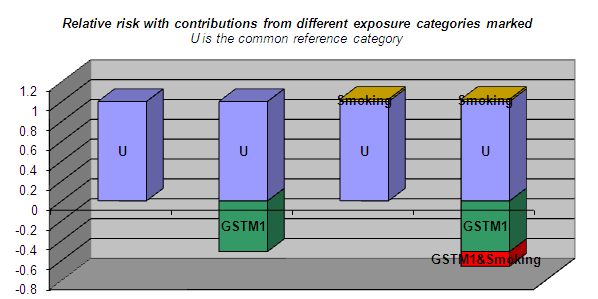


**Graphs showing an example of relative risk with contributions from different exposure categories.** A: Interaction between GSTP1 341C/C+T/T genotype with wood/charcoal; B; interaction between *GSTM1*0/*0* genotype with smoking. U is the reference which is GSTP1 341C/C and none exposure to wood/charcoal (**A)** and *GSTM1*1/*1* and non-smokers (B) according to Andersson et al. [39].
